# Psychometric evaluation of the full and shortened versions of the WGCTA-II in Slovak university students

**DOI:** 10.3389/fpsyg.2026.1764712

**Published:** 2026-02-02

**Authors:** Gabriela Šeboková, Lucia Ráczová, Jana Uhláriková, Ema Lukačková, Tomáš Sollár

**Affiliations:** Department of Psychological Sciences, Faculty of Social Sciences and Health Care, Constantine the Philosopher University in Nitra, Nitra, Slovakia

**Keywords:** confirmatory factor analysis, critical thinking, item response theory, psychometric evaluation, Slovak university students, WGCTA-II

## Abstract

**Introduction:**

Critical thinking (CT) is a key cognitive skill essential for academic and informed decision-making. Although the Watson-Glaser Critical Thinking Appraisal (WGCTA) has been widely used internationally, its psychometric properties have not yet been systematically evaluated in Slovakia. The present study aimed to examine the reliability and validity of the Slovak WGCTA-II (Form C) and to develop a shortened version using contemporary psychometric methods.

**Method:**

Slovak university students (*N* = 264) completed the WGCTA-II and two versions of the Cognitive Reflection Test (CRT-V, CRT-N) for criterion validity. Reliability analyses, confirmatory factor analyses (CFA), parallel analysis, and Item Response Theory (IRT) models were used to examine internal consistency, dimensionality, and item functioning. Based on theoretical relevance and psychometric performance, two core dimensions—Interpretation and Evaluation of Arguments—were retained. An independent sample (*N* = 137) was used to replicate reliability and model fit.

**Results:**

The original WGCTA-II showed acceptable overall reliability but limited construct validity at the subscale level. The resulting 9-item unidimensional version demonstrated good IRT model fit (RMSEA = 0.042; SRMR = 0.076) and satisfactory reliability (*ω* = 0.66), replicated in the second sample. Criterion validity was supported by correlations with the Cognitive Reflection Test (*r* = 0.30–0.38).

**Discussion:**

These findings provide the first psychometric evidence for the Slovak WGCTA-II, demonstrate the utility of combining CTT and IRT for robust test evaluation, and introduce a concise, culturally adapted tool for efficient assessment of critical thinking, contributing to methodological innovation in psychological measurement.

## 1 Introduction

Critical thinking (CT) is widely recognized as a core 21st-century skill and a key predictor of success in education and society (Voogt and Roblin, 2012; Rusmin et al., 2024; Rothinam et al., 2025). This is largely because CT is associated with key competencies for effective functioning in contemporary society, such as informed decision-making (Stupple et al., 2017; Ren et al., 2020), employability, and civic engagement (Facione and Facione, 2001; Minnameier and Hermkes, 2020; Simonovic et al., 2022). Moreover, CT is increasingly viewed as a central outcome of higher education, underscoring the importance of fostering this ability among university students, whose academic achievement has also been shown to be linked to CT (Rivas et al., 2023).

Although many definitions of CT have been proposed (e.g., Ennis, 2011; Halpern, 2014; Moore, 2011), a particularly comprehensive one was formulated by the American Philosophical Association Delphi panel of 46 experts, defining critical thinking as “purposeful, self-regulatory judgment which results in interpretation, analysis, evaluation, and inference, as well as explanation of the evidential, conceptual, methodological, criteriological, or contextual considerations upon which that judgment is based “(Facione, 1990, p. 3). Although CT may manifest differently across contexts and disciplines, a shared feature across conceptualizations is the ability to analyze and evaluate arguments and the evidence supporting them – skills widely recognized as central to critical reasoning (Dwyer et al., 2014; Mueller et al., 2020; Liu et al., 2014; Stanovich et al., 2016). Despite variations in definitions, this common emphasis supports viewing CT as a coherent construct centered on analytic and evaluative reasoning.

Given its importance, the accurate assessment of CT is essential for both research and educational practice. Although ministries of education worldwide emphasize the development of CT, only a limited number of assessment tools have been validated for use in different populations (Hassan and Madhum, 2007). Several standardized instruments exist, including the Watson–Glaser Critical Thinking Appraisal (WGCTA; Watson and Glaser, 1980), the Cornell Critical Thinking Test (Ennis and Millman, 1985), the California Critical Thinking Skills Test (Facione, 1990), and the Halpern Critical Thinking Assessment (Halpern, 2010). Despite their widespread use, concerns remain regarding their psychometric quality. Research has revealed inconsistent evidence of validity and reliability, including low reliability coefficients (Abrami et al., 2008; Verburgh et al., 2013), unstable factor structures (Abrami et al., 2008; Bernard et al., 2008; Leach et al., 2020; Verburgh et al., 2013), problematic response formats (Bernard et al., 2008; Ku, 2009), ambiguous instructions (Fawkes et al., 2003; Possin, 2014), limited research applications (Liu et al., 2014) and cross-cultural equivalence (Butler et al., 2012). A persistent challenge in the critical thinking literature concerns how to validly assess such a multifaceted construct, further complicated by the limited empirical evidence supporting the correspondence between test items and their underlying theoretical dimensions (Leach et al., 2020). These issues highlight the need for thorough psychometric evaluation of CT assessments in diverse populations. The present study therefore focuses on the Watson–Glaser Critical Thinking Appraisal (WGCTA)—the oldest, most widely used, and most extensively studied CT test (Bernard et al., 2008). Although the WGCTA has been applied across a wide range of educational and professional contexts and is valued for assessing core components of CT (Wayas et al., 2024; Afzal et al., 2024; Šeboková et al., 2025), its continued use depends on robust evidence of its psychometric adequacy, making its evaluation both theoretically and practically important.

The WGCTA defines critical thinking as “the ability to identify and analyze problems, seek and evaluate relevant information, and reach an appropriate conclusion” (Watson and Glaser, 2018). It assesses five interrelated skills in a verbal context: (1) Inference – judging the likelihood that conclusions follow from given information; (2) Recognition of assumptions – recognizing implied assumptions or presuppositions behind the provided statements; (3) Deduction – evaluating whether conclusions logically follow from premises; (4) Interpretation – assessing whether conclusions are justified by evidence; and (5) Evaluation of arguments – determining the strength and relevance of arguments (Watson and Glaser, 2018). Since its introduction, the WGCTA has undergone several revisions. The original 1964 version included 100 items (Forms Ym and Zm). In 1980, Forms A and B (WGCTA-II, 80 items) were published, followed by a UK adaptation of Form B (Form C; Rust, 2002). A 40-item Short Form S and later parallel Forms D and E were also developed. The most recent revision, WGCTA-III (Watson and Glaser, 2018), introduced updated, business-oriented items and is now available in an online format.

Although the WGCTA has been extensively studied internationally, its psychometric properties have not yet been systematically examined in Slovakia. The Slovak adaptation of WGCTA-II (Form C) exists (Watson and Glaser, 2000), but no published data are available regarding its reliability, validity, or factor structure in Slovak university students. Moreover, the administration of the WGCTA-II requires approximately 40 min, which may limit its feasibility for large-scale research applications. Examining the Slovak version of the WGCTA-II in this context provides valuable empirical evidence on its psychometric soundness and practical applicability, thereby contributing to a broader understanding of the cross-cultural validity of critical thinking assessment tools, specifically in central-eastern European context.

Over the years, numerous studies have examined the psychometric properties of various WGCTA versions, highlighting concerns about their validity and reliability, particularly regarding the factor structure of the test. Some studies suggest a unidimensional solution rather than separate scores for the five subscales (Bernard et al., 2008; Hassan and Madhum, 2007). Bernard et al. (2008) examined 60 sets of subscale means and 13 sets of intercorrelations reported in published studies with diverse learner groups, identifying a consistent one-factor solution and concluding that the WGCTA measures a general critical thinking skill rather than five distinct dimensions. Similar findings emerged from a study with Lebanese university students, where exploratory factor analysis of the WGCTA-S also indicated a unidimensional structure (Hassan and Madhum, 2007). Taken together, these findings challenge the theoretical assumption of five separable dimensions and raise questions about the construct validity of the WGCTA subscales.

Another concern involves the wide range of reliability coefficients, reported between 0.23 and 0.73 (Bernard et al., 2008) or 0.17 to 0.74 (Loo and Thorpe, 1999). This variability suggests that the reliability of the WGCTA is not a stable property of the instrument but may be strongly context dependent. Previous research suggests that reliability may be influenced by contextual differences across learners and settings (Bernard et al., 2008), the multiple-choice format that increases the likelihood of guessing (Wagner and Harvey, 2006), and challenges related to cross-cultural applicability and face validity. Additional limitations of the WGCTA include non-comparable test forms, unclear evidence of differential validity across respondent groups, and limited evidence of incremental validity (Liu et al., 2014). Together, these issues limit the interpretability and comparability of WGCTA scores across studies and raise concerns about its suitability for cross-cultural research without thorough local validation.

Despite these issues, some studies provide evidence of convergent and criterion validity. For instance, WGCTA-S scores moderately correlated with analysis and problem-solving (*r* = 0.52) and judgment and decision-making (*r* = 0.52) (Ejiogu et al., 2006), as well as with undergraduate course grades in psychology and education (*r* = 0.20–0.62) (Gadzella et al., 2006). Overall, the available evidence indicates that while the WGCTA demonstrates meaningful convergent and criterion validity, its limitations are primarily related to the psychometric functioning of specific items and subscale structures, pointing to the need for systematic refinement rather than rejection, which subsequently motivated revisions of the test.

In response to these considerations, the Red Model was introduced in WGCTA-II, reorganizing items into a simplified three-factor structure: Recognize Assumptions, Evaluate Arguments, and Draw Conclusions (combining Inference, Deduction, and Interpretation). Confirmatory factor analysis supported this structure, with internal consistency ranging from 0.81 to 0.89 (NCS Pearson, Inc., 2009). Convergent and criterion validity were demonstrated through correlations with WAIS-IV (*r* = 0.52), Raven’s APM (*r* = 0.53), and the Advanced Numerical Reasoning Appraisal (*r* = 0.68). The RED model formed the basis for WGCTA-III, which features a new, globally applicable item bank. However, the RED model and its three-factor structure have not yet been independently and extensively evaluated. This gap in empirical evidence constrains both the measurement and conceptual understanding of critical thinking within educational settings. As Ku (2009) noted in her review of the psychometric properties of CT assessments, test developers and affiliated researchers tend to report more favorable psychometric qualities than independent researchers. This underscores the need for more impartial, externally conducted validations.

Moreover, no comprehensive item-level psychometric investigation has been conducted. IRT has several advantages over the classical test theory (CTT). In CTT, reliability is considered a property of the entire test, typically measured using coefficients such as Cronbach’s alpha. This approach necessitates re-evaluation if the test undergoes modifications, such as shortening or adaptation. In contrast, Item Response Theory (IRT) evaluates reliability at the item level, providing a more precise analysis of how each question contributes to assessing critical thinking, thereby enhancing the accuracy and flexibility of the measurement (Bond, 2015). IRT provides an accurate and consistent measurement of CT by accounting for both item difficulty and individual ability. Unlike traditional methods, it separates ability from item characteristics, ensuring more valid and reliable estimates while maintaining fairness through probabilistic modeling, where higher-ability students are more likely to answer difficult questions correctly (Bond, 2015). Critical thinking comprises multiple subscales, necessitating measurement tools that can assess each ability separately. Empirical research indicates that using the item response theory approach enhances the precision of cognitive and complex thinking skill assessments by providing a more detailed analysis of an instrument’s reliability and validity (Suwita et al., 2024). Therefore, this methodological framework seems suitable for present study.

Given the unresolved issues with the WGCTA – unclear factor structure, inconsistent reliability, cultural applicability concerns, and the absence of item-level psychometric investigations—further examination is needed. In addition, the test’s considerable length and limited large-scale research applications in higher education contexts point to the need for a shorter yet psychometrically sound version. Building on the limitations, the present study had three main aims. First, we examined the psychometric properties of the Slovak version of the WGCTA – II (Form C) in a sample of university students. Specifically, we conducted reliability analyses and confirmatory factor analyses to clarify the underlying structure of the test’s five subscales. This step was necessary to determine whether the original multidimensional structure of the WGCTA would be empirically supported in our cultural context and to identify potential sources of psychometric weakness. Second, based on the results of the factor analyses and the item-level diagnostics, we performed an item response theory (IRT) analysis using the Rasch model to evaluate a shortened version of the WGCTA that would retain only the most informative and psychometrically valid items. The goal was to develop a more practical and efficient measure of critical thinking suitable for research and educational settings, without compromising reliability or validity. Third, we examined the reliability and the convergent validity of the shortened version of WGCTA on two samples of university students.

## 2 Methods

### 2.1 Research sample

The research sample consisted of 266 university students enrolled in bachelor’s degree programs at two universities in Slovakia. Two participants were excluded due to incomplete data, resulting in a final sample of 264 respondents (168 men, 96 women; *M_age_* = 19.94, *SD_age_* = 1.36). Of these, 216 students were in their first year and 48 students were in their second year. The sample included 40% students from the humanities (psychology) and 60% students from technical fields (automatic production, control systems). Most participants (79%) were of Slovak nationality.

The second research sample comprised 137 undergraduate students (32 men, 105 women; *M_age_* = 20.93, *SD_age_* = 1.45) from universities in Slovakia. This sample included 69% students from the humanities (psychology and social work) and 31% students from technical fields (landscape engineering) studying at two Slovak universities. Most participants (98%) were of Slovak nationality. Regarding academic year, 120 students were in their second year and 17 were in their third year.

### 2.2 Materials

*Watson–Glaser Critical Thinking Appraisal* (WGCTA-II, form C; Watson and Glaser, 1991): The WGCTA contains statements representing a wide range of written and spoken materials commonly encountered in everyday situations and exists in several versions, including updated editions used internationally. For the purposes of this study, we employ the version that is currently the only standardized version available in our context, as more recent editions, although existing abroad, have not yet been adapted or validated locally. The test comprises 80 items divided into five subscales (each consisting of 16 items): Inference – the ability to judge whether conclusions follow from the provided information. Recognition of Assumptions – the ability to identify implicit assumptions in statements. Deduction – the ability to determine whether a conclusion logically follows from given premises. Induction/Interpretation – the ability to evaluate whether evidence and conclusions can be generalized. Evaluation of Arguments – the ability to assess the relevance and strength of arguments related to a problem. For the Inference subscale, a 5-point Likert scale is used, whereas the other four subscales employ a dichotomous (1/0) response format. The test includes distinct instructions and practice questions with answers for each of the five subscales to help participants understand the task and familiarize themselves with the test format. This structure makes the test cognitively and procedurally demanding, as participants must undergo brief training or familiarization before each subtest.

Example of the statement: Research on vocabulary development in children from 8 months to 6 years has shown that the number of words used grows from 0 words at 8 months to 2,562 words at 6 years.

None of the children who participated in the study could speak at 6 months (mark YES, the conclusion follows unequivocally from the statement, since the vocabulary of children at 6 months was 0 words).

The growth of vocabulary is slowest during the period when children learn to walk (mark NO, the conclusion does not follow from the statement, as there is no information about the relationship between vocabulary and learning to walk).

*Cognitive Reflection Test* (CRT): Convergent validity of the WGCTA (both original and shortened) was evaluated using both the numerical (Frederick, 2005) and verbal (Sirota et al., 2020) versions of the Cognitive Reflection Test. The CRT assesses the ability to suppress intuitive but incorrect responses in favor of reflective, deliberative reasoning, based on the dual-processing framework of cognition. Type 1 processing is fast and intuitive, often leading to biased responses, whereas Type 2 processing is slower, reflective, and supports logical reasoning (Frederick, 2005; Stanovich et al., 2016). Critical thinking is conceptually related to Type 2 processing, as reflective thinking facilitates the development and application of rules and strategies necessary for analytical problem solving (Simonovic et al., 2022; Bonnefon, 2018). Including both versions allows assessment of cognitive reflection while accounting for potential confounding with numeracy skills in the numerical version and focusing on reasoning ability in the verbal version (Sirota et al., 2020). This approach ensures a comprehensive and theoretically relevant measure for convergent validation of critical thinking.

*Numerical CRT* (Frederick, 2005): The test consists of 3 items (e.g., “If it takes 3 people 3 min to make 3 products, how long would it take 10 people to make 10 products?”). Each item has two response alternatives: one representing intuitive thinking and one representing reflective thinking. Participants select one answer, with no time limit imposed. Scores range from 0 (no correct answers) to 3 (all correct), with higher scores indicating greater cognitive reflection.

*Verbal CRT* (Sirota et al., 2020): The test includes 10 open-ended problems (e.g., “Laura’s father has 5 daughters but no sons: Nana, Nene, Nini, Nono. What is the fifth daughter’s name?”). It measures the ability to suppress a default intuitive response in favor of a correct reflective answer. Scores range from 0 (no correct answers) to 10 (all correct), with higher scores indicating greater cognitive reflection.

### 2.3 Procedure

Data for the first research sample were collected during the winter term of 2023/2024 under supervised, paper-and-pencil conditions. Participants received oral and written feedback on their test scores, and individual reports were provided upon request via email or post. Data for the second research sample were collected in the winter term of 2024/2025, also under supervision. Participants again received both oral and written feedback, and individual reports were emailed upon request.

All procedures followed were in accordance with the ethical standards of the responsible committee on human experimentation (both institutional and national) and with the Declaration of Helsinki of 1975, as revised in 2000. The study was also approved as part of an ongoing project (VEGA 1/0336/24: Critical thinking in relation to academic success and decision-making in specific areas of students´ lives) by the ethics committee (UKF/370/2025/191013:024).

### 2.4 Statistical analysis

All analyses were conducted in R (Version 4.2.2; R Core Team, 2022). Data preparation and descriptive statistics were performed using the dplyr package (Wickham et al., 2023a) and tidyr (Wickham et al., 2023b), while visualizations were created with ggplot2 (Wickham, 2016). One item with zero variance (WG_ Assumptions _31) was excluded prior to all analyses.

Internal consistency was evaluated using Cronbach’s *α* and McDonald’s *ω*, computed from the polychoric correlation matrix due to the dichotomous scoring. Reliability indices, item difficulties, and item–total correlations were obtained using psych (Revelle, 2023) in combination with polycor (Fox, 2019).

The dimensional structure of each WGCTA subscale was examined using confirmatory factor analysis (CFA) with a single-factor model per subscale. Our analytic strategy followed a stepwise logic. First, we examined the unidimensionality and fit of each WGCTA subscale separately using first-order CFA models. This approach was necessary to evaluate whether the basic assumptions for testing a higher-order or hierarchical model were met. Given the inadequate fit and low factor loadings observed at the subscale level, higher-order CFA models were not pursued. CFAs were estimated with the WLSMV estimator in lavaan (Rosseel, 2012), with additional utilities provided by semTools (Jorgensen et al., 2022). Model fit was evaluated using χ^2^, RMSEA, CFI, TLI, and SRMR, following recommended APA and structural-equation-modeling guidelines (Hu and Bentler, 1999; Kline, 2016). Values of CFI and TLI ≥ 0.90 indicate acceptable fit, and ≥ 0.95 indicate excellent fit. RMSEA < 0.06 and SRMR < 0.08 are generally regarded as indicative of good fit. Because most subscales demonstrated inadequate fit (e.g., low CFI/TLI), higher-order WGCTA models were not tested.

To develop a shortened WGCTA version, items from the Interpretation and Evaluation of Arguments subscales were selected using parallel analysis (via psych, Revelle, 2023; TAM, Robitzsch et al., 2021) and factor loadings.

The shortened version was evaluated using a Rasch model estimated with mirt (Chalmers, 2012). Model fit was assessed using χ^2^ fit statistics, RMSEA, SRMR, infit/outfit, and S-X^2^ indices. Criterion validity was examined via correlations with CRT-N and CRT-V using psych (Revelle, 2023). Differences between dependent correlations were tested with Steiger’s Z test using cocor (Diedenhofen and Musch, 2015). Finally, the nine-item Rasch model was replicated on an independent sample using the same analytic procedures to confirm model stability, item functioning, and criterion validity.

## 3 Results

### 3.1 Psychometric analysis of the full WGCTA-II and its individual dimensions

The analyses were conducted on 79 items of the Slovak version of the WGCTA, covering five subscales: Inference, Recognition of Assumptions, Deduction, Interpretation, and Evaluation of Arguments. One item with zero variance (WG_Assumptions_31) was excluded. All items were scored dichotomously (0 = incorrect, 1 = correct).

Item difficulty varied widely across the WGCTA subscales (see Supplementary Table 1). Inference was the most challenging, Recognition of Assumptions the easiest, Deduction showed moderate difficulty, Interpretation ranged from easy to difficult items, and Evaluation of Arguments exhibited relatively consistent medium difficulty.

Internal consistency was assessed using Cronbach’s alpha and McDonald’s omega coefficients. The overall WGCTA showed moderate to good reliability (*α* = 0.691; *ω* = 0.846). However, it is important to note that both Cronbach’s alpha and McDonald’s omega are sensitive to the number of items, and higher values may reflect test length rather than true inter-item homogeneity (Cortina, 1993; Raykov, 1997). Therefore, the relatively high reliability of the total WGCTA score should not be interpreted as evidence of a well-functioning or internally coherent multidimensional structure. In contrast, reliability estimates for the individual subscales were substantially lower (*α* = 0.23–0.51; ω = 0.49–0.72; see Table 1), indicating weak internal consistency and limited homogeneity of items within the proposed dimensions. This discrepancy between the relatively higher reliability of the total score and the poor reliability of the individual subscales suggests that the original multidimensional structure of the WGCTA is not psychometrically coherent in the present sample.

**Table 1:** Internal consistency coefficients for the total scale and subscales of the WGCTA-II (full version).

Subscale	Number of items	Cronbach’s α	McDonald’s *ω*
Total WGCTA	79	0.691	0.846
Inference	16	0.469	0.594
Assumptions	15	0.262	0.497
Deduction	16	0.234	0.654
Interpretation	16	0.491	0.719
Arguments	16	0.511	0.667

Given these findings, subsequent analyses focused on evaluating the dimensional structure of each WGCTA subscale separately before testing the overall model. This stepwise approach is crucial, as assessing individual subscales provides insight into whether they represent coherent latent dimensions. If the subscales fail to demonstrate adequate internal consistency or unidimensionality, testing a comprehensive higher-order model would not be theoretically or empirically justified. Accordingly, a separate confirmatory factor analysis (CFA) was conducted for each WGCTA subscale, testing a single-factor model for each dimension. The results (Table 2) indicated that the single-factor model showed poor fit across most subscales, with CFI and TLI values well below the recommended threshold of 0.90 and in some cases even below 0.70, suggesting that a unidimensional solution was inadequate. The best fit was observed for the Interpretation subscale, whereas Recognition of Assumptions and Evaluation of Arguments showed the poorest fit. The standardized factor loadings of the items are presented in Supplementary Table 2. Inspection of the standardized factor loadings revealed that a substantial proportion of items across most subscales exhibited loadings below commonly recommended thresholds (e.g., < 0.30), with several items showing very weak or negative loadings. This pattern indicates limited association between many indicators and their intended latent dimensions and suggests that the original subscale structure is not well supported at the item level in the present sample. Given that even the individual subscales did not demonstrate satisfactory fit, we did not proceed to test a higher-order model across the full WGCTA. Testing a higher-order CFA model would require adequate unidimensionality and acceptable fit at the subscale level. Given that most subscales failed to meet these prerequisites, proceeding with a second-order model would have been both theoretically and statistically unjustified.

**Table 2:** Confirmatory factor analysis of the WGCTA-II subscales (full version).

Subscale	χ^2^	df	*p*	RMSEA	CFI	TLI	SRMR
Inference	158.29	104	< 0.001	0.045	0.746	0.707	0.112
Assumptions	138.81	90	0.001	0.045	0.649	0.590	0.118
Deduction	230.91	104	< 0.001	0.068	0.767	0.731	0.121
Interpretation	181.02	104	< 0.001	0.053	0.809	0.780	0.121
Arguments	230.40	104	< 0.001	0.068	0.603	0.542	0.129

### 3.2 Item selection for the shortened WGCTA version

The results of the analyses indicated that the items of the original WGCTA did not contribute evenly to the measurement of the critical thinking construct, with several dimensions showing low reliability and weak construct validity. Accordingly, the second aim of the study was to develop a more efficient and reliable WGCTA version by selecting items with suitable difficulty, higher factor loadings, and positive contributions to internal consistency.

Given the large number of items, we first selected subscales that best represented the critical thinking construct, combining theoretical considerations (RED model) with empirical results. From the Draw Conclusions dimension, only the Interpretation subscale was retained due to superior internal consistency and fit, while Inference and Deduction were excluded for low reliability, poor fit, high difficulty, and complex item formats. Evaluation of Arguments was retained despite lower CFI and TLI values because of acceptable RMSEA, adequate reliability (*ω* = 0.667), and balanced item difficulty; Recognition of Assumptions was excluded due to very low reliability and negative factor loadings. Parallel analysis revealed that many indicators within the Interpretation and Evaluation of Arguments subscales failed to demonstrate sufficiently strong associations with their latent dimensions, as reflected in standardized factor loadings below commonly recommended thresholds (e.g., < 0.30). Parallel analysis within Interpretation and Evaluation of Arguments identified the most psychometrically and content-relevant items, resulting in a 9-item shortened scale (5 Interpretation, 4 Evaluation of Arguments; see Table 3). Items with low factor loadings (below 0.25) and negative item-total correlations were excluded to improve construct validity. The selection combined empirical criteria (loadings, model fit, reliability indices) with theoretical relevance, ensuring that retained items represent the core critical thinking processes. Item selection also accounted for linguistic and cultural appropriateness (e.g., avoiding double negatives and culturally specific expressions) and ensured a balance between strong and weak arguments as well as correct and incorrect answers. Low factor loadings were interpreted not as isolated statistical artifacts, but as systematic indicators of weak construct representation, cultural mismatch, or excessive cognitive and linguistic complexity of certain items. The final set meets psychometric quality standards while encompassing key aspects of critical thinking, such as evidence interpretation and argument evaluation.

**Table 3:** Paralel analysis of interpretation and arguments subscales.

Item	Loading
Interpretation	
WG_interpretation_51	0.548
WG_interpretation_56	0.485
WG_interpretation_53	0.420
WG_interpretation_54	0.414
WG_interpretation_61	0.403
WG_interpretation_60	0.401
WG_interpretation_49	0.305
WG_interpretation_62	0.304
WG_interpretation_63	0.246
WG_interpretation_59	0.237
WG_interpretation_55	0.216
WG_interpretation_50	0.044
WG_interpretation_58	0.040
WG_interpretation_64	0.024
WG_interpretation_57	−0.054
WG_interpretation_52	−0.153
Arguments	
WG_arguments_69	0.452
WG_arguments_71	0.451
WG_arguments_78	0.361
WG_arguments_68	0.330
WG_arguments_66	0.306
WG_arguments_80	0.287
WG_arguments_76	0.285
WG_arguments_79	0.280
WG_arguments_67	0.263
WG_arguments_65	0.237
WG_arguments_70	0.210
WG_arguments_73	0.174
WG_arguments_75	0.151
WG_arguments_74	0.113
WG_arguments_72	0.103
WG_arguments_77	0.020

### 3.3 Examination of the shortened WGCTA version using IRT

The correlation between the Interpretation and Evaluation of Arguments dimensions was 0.48, indicating a moderate relationship. This level of correlation led to the consideration of a common factor and the development of a unidimensional scale. The nine-item scale was subsequently evaluated using IRT analysis with the Rasch model. The results indicated that the nine-item model demonstrated an acceptable fit to the data (χ^2^(35) = 50.996, *p* = 0.039; RMSEA = 0.042; SRMR = 0.076). RMSEA values below 0.06 and SRMR values below 0.08 suggest good model fit (Hu and Bentler, 1999), while the chi-square test result points to only minor deviations, which are acceptable given the small number of items.

The 9-item scale demonstrated moderate internal consistency (*α* = 0.65; *ω* = 0.66), which is acceptable given the shortened length and suitable for practical use. Item difficulty estimates ranged from −2.40 (WG_interpretacia_54) to 0.41 (WG_interpretacia_62; Table 4), indicating that the combination of the most psychometrically and content-relevant items from both subscales produced a unidimensional 9-item scale with adequate fit and reliability.

**Table 4:** Item difficulty estimates for the shortened WGCTA-II based on the Rasch model.

Item	Difficulty (b)
WG_interpretation_51	−1.119
WG_interpretation_54	−2.403
WG_interpretation_56	−1.051
WG_interpretation_60	−1.813
WG_interpretation_62	0.407
WG_arguments_68	−1.306
WG_arguments_69	−1.506
WG_arguments_71	−1.532
WG_arguments_79	−0.631

### 3.4 Criterion validity of the shortened WGCTA version

Criterion validity was assessed by correlating the total scores of the shortened and original WGCTA with CRT performance. The nine-item version showed moderate correlations with CRT-N (*r* = 0.381) and CRT-V (*r* = 0.299), comparable to those of the original version (*r* = 0.329 and *r* = 0.298, respectively).

Differences between the correlations of the shortened and full WGCTA versions with external criteria were tested using Steiger’s test (Steiger, 1980), which accounts for the dependency between correlations sharing a common variable. Results indicated no statistically significant differences, suggesting that the shortened version preserves the criterion validity of the full test.

### 3.5 Replication of the shortened WGCTA in an independent sample

The nine-item unidimensional model was also validated on an independent sample of university students using the Rasch model. The model demonstrated good fit to the data (χ^2^(35) = 40.768, *p* = 0.231; RMSEA = 0.035; SRMR = 0.09) and high reliability (*ω* = 0.70). Infit and outfit values for all items fell within the acceptable range of ±2, confirming the adequate fit of individual items. Furthermore, S-X^2^ tests and item characteristic curves indicated an even distribution of the probability of correct responses across the latent trait range (−2 to +2), supporting the suitability of the items for the shortened scale.

Criterion validity was also examined by correlating the shortened WGCTA with CRT-N (*r* = 0.303, *p* < 0.001) and CRT-V (*r* = 0.307, *p* < 0.001). The correlations were moderate and consistent with the results from the first data collection, further supporting the criterion validity of the nine-item version on an independent sample.

## 4 Discussion

The present study examined the psychometric properties of the Slovak version of the WGCTA-II (form C) in a sample of university students and evaluated a shortened version that maintains psychometric rigor while offering greater practical feasibility. Specifically, the study assessed whether the original structure of the WGCTA is supported in a new cultural and linguistic context and sought to identify potential sources of measurement instability at both the subscale and item levels. By integrating empirical analyses and theoretical considerations, we aimed to provide a reliable and efficient tool for both research and educational applications.

Across multiple levels of analysis, the results consistently indicated limited support for the intended multidimensional structure, including poor model fit of several subscales, weak and unstable item–factor relationships, and low internal consistency at the dimension level. From a measurement perspective, these findings also precluded meaningful testing of higher-order or hierarchical CFA models, as the prerequisite unidimensionality at the subscale level was not met. Although the total WGCTA score demonstrated acceptable reliability, this result should be interpreted with caution, as it appears to be driven primarily by test length rather than by coherent measurement of distinct components of critical thinking. In contrast, the convergence of low subscale reliability, inadequate factor loadings, and inconsistent dimensional fit raises substantial concerns regarding the construct validity of the original subscale configuration. The findings point to a broader pattern of psychometric weaknesses in the full Slovak version of the WGCTA-II, and together with its considerable time demands and complex administration, suggest that it is not optimal for research purposes. This conclusion aligns with previous findings (e.g., Bernard et al., 2008; Ku, 2009; Liu et al., 2014; Verburgh et al., 2013), and reflects the broader challenges associated with validating the factorial structure of instruments designed to measure complex constructs such as critical thinking, particularly when individual items are intentionally constructed to engage multiple, interrelated cognitive processes rather than discrete abilities (Leach et al., 2020).

Current trends in critical thinking research point toward the development of shorter, psychometrically robust, and culturally adapted instruments (Payan-Carreira et al., 2022; Rivas et al., 2023; Fabio et al., 2025; Butler et al., 2012). Test shortening allows for more efficient assessment while maintaining validity and reducing the administrative burden on both participants and researchers. In line with this approach, we focused on developing a shortened version of the WGCTA that combines conceptually key dimensions and empirically validated items while preserving psychometric quality.

In selecting the most appropriate indicators of critical thinking, we applied a combination of theoretical and empirical criteria (DeVellis, 2017; Kline, 2016). Rather than attempting to preserve the original five-subscale structure at all costs, we adopted a parsimonious approach that prioritized dimensions demonstrating both empirical stability and conceptual centrality to critical thinking. The empirical criteria included factor loadings, model fit, and reliability indices, while the theoretical criteria were derived from the RED model of critical thinking and considered the content validity. From the construct perspective, we retained two dimensions—Interpretation and Evaluation of Arguments—which represent the core processes of critical thinking. The Interpretation dimension reflects the ability to assess the reliability and relevance of evidence and to avoid common cognitive biases such as reasoning fallacies, jumping to conclusions, confusing correlation with causation, or the indefinite pronoun fallacy (Stanovich and West, 2008; Kahneman, 2011). The Evaluation of Arguments dimension complements Interpretation by focusing on the assessment of the logical strength of claims and the identification of manipulative or insufficiently supported arguments. These dimensions showed comparatively higher internal consistency, more interpretable factor loadings, and more balanced item difficulty profiles than the remaining subscales.

The selection of these two dimensions is also consistent with broader theoretical conceptualizations of critical thinking (e.g., Facione, 1990; Halpern, 2014). The core skills identified by the widely recognized Delphi panel – analysis, evaluation, and inference – align with our dimensions, as they involve recognizing argument structure, assessing credibility, and evaluating evidence. Similarly, Reflective judgment theory (King and Kitchener, 2004), the Dual Process Model (Stanovich et al., 2016) and Kuhn’s framework of argumentative reasoning (Kuhn, 1991) emphasize analytic and evaluative reasoning as central components of critical thinking. Taken together, this body of evidence indicates that interpretation and evaluation of arguments constitute the core of critical thinking across theoretical models and represent fundamental skills for both academic and civic competence, internationally (Van Gelder, 2012; Liu et al., 2014; Mueller et al., 2020; American Psychological Association, 2013, 2016; Yulian, 2021) as well as within the Slovak context (Research Team, 2018; Kotlebová and Hankerová, 2024). In today’s information-rich environment, these two processes are critical for accurate reasoning and judgment, whereas other components, though supportive, do not form the core of critical thinking.

The only RED Model dimension not included in the final version was Recognizing Assumptions. Although this dimension – the ability to distinguish facts from opinions and identify implicit beliefs – represents a relevant but not central component of critical thinking, its psychometric performance was unsatisfactory. The items showed low internal consistency and limited contribution to the overall construct, failing to adequately differentiate between individuals with higher and lower levels of critical thinking. This pattern is consistent with theoretical perspectives on paradigmatic assumptions, which suggest that deeply held assumptions are often implicit and difficult to access through self-report or decontextualized questionnaire formats, thereby limiting the sensitivity of such items to individual differences (Brookfield, 2015).

Moreover, item selection also considered content and contextual appropriateness, in addition to statistical indicators. Items requiring work with evidence, identification of logical fallacies, and evaluation of argumentative strength were prioritized, while less suitable items were excluded due to culturally specific contexts, ambiguous wording, or excessive linguistic complexity. A balanced representation of items with correct and incorrect answers was maintained to support content balance and measurement fairness. These results highlight the importance of culturally sensitive adaptation, as even originally validated items may function differently across linguistic and cultural contexts (Butler et al., 2012; Fabio et al., 2025). In addition, findings underscore that psychometric quality cannot be separated from cultural and linguistic appropriateness, highlighting the necessity of careful adaptation when applying standardized critical thinking measures across contexts.

The results of the IRT analysis indicated that selecting items that were both psychometrically and content-wise strong produced a unidimensional model with a reliable and valid structure, confirmed in an independent replication sample. Conceptually, interpretation and evaluation of arguments represent distinct but closely related processes of critical thinking. Empirically, however, their strong interrelation within the psychometrically refined item set resulted in a unidimensional measurement model in the shortened WGCTA. Importantly, the unidimensionality should not be interpreted as evidence that critical thinking itself is a single, undifferentiated ability, but rather as a property of the selected measurement model. This distinction is crucial, as unidimensionality at the measurement level does not preclude multidimensionality at the conceptual level and is consistent with theoretical and empirical perspectives that conceptualize critical thinking as a system of interrelated core processes (Bernard et al., 2008; Dwyer et al., 2014; Grimm and Richter, 2024; Fabio et al., 2025; Rivas et al., 2023). Given the limited fit and instability of multi-factor solutions observed in the present data, the unidimensional model provided the most robust and interpretable representation.

The relationship between the shortened version and the Cognitive Reflection Test (CRT) provided additional evidence of criterion validity. A moderate relationship suggests that the two tests capture related, yet not identical, processes. While the CRT focuses on the inhibition of intuitive responses (System 1), the WGCTA reflects engagement of System 2 as well as broader analytical and argumentative skills. The ability to stop and start logical processing of information can be considered a prerequisite for CT. In this context, cognitive reflection may reflect a core cognitive mechanism underlying critical thinking, whereas the WGCTA captures the broader set of reasoning and evaluative skills, indicating a complementary, rather than redundant, relationship between the two measures. By examining this relationship across both the original and shortened WGCTA versions and replicating the findings in an independent sample, the present study further demonstrates the robustness, generalizability, and preservation of the theoretical and empirical properties of the shortened form (Furr, 2021).

### 4.1 Limitation

The present study has several limitations. For the purposes of this research, we employed the version of the WGCTA that is currently the only standardized version available in our context, as more recent editions, although available internationally, have not yet been adapted or validated locally. Consequently, the results of this study are limited to this older version, which should be considered when interpreting the findings. Second, the shortened version of the test focuses on key, but not all, dimensions of critical thinking. Therefore, the results should be interpreted as a screening indicator of critical thinking, primarily suitable for research purposes, rather than as a comprehensive diagnostic assessment. Third, criterion validity was assessed only in relation to the CRT. Future studies should include additional indicators of criterion validity, such as other tests or performance-based tasks. It would also be valuable to examine the predictive validity of the test in relation to students’ cognitive and academic outcomes and to determine the extent to which it captures changes in critical thinking following specific courses or training interventions. Furthermore, it would be beneficial to investigate how the test performs across different academic disciplines, age groups, or in online settings, where critical thinking may manifest differently than in traditional testing situations.

## 5 Conclusion

Despite these limitations, the validated and reliable 9-item version of the WGCTA provides a practical and culturally adapted tool for the rapid assessment of critical thinking in higher education. Its shortened format reduces administration time and cognitive load, making it suitable for large-scale research, course evaluation, and studies examining instructional interventions aimed at developing critical thinking. For educational institutions, it offers an accessible means of monitoring the development of a key 21st-century competency, while maintaining sufficient psychometric quality for research and applied assessment contexts.

## Data Availability

The raw data supporting the conclusions of this article will be made available by the authors, without undue reservation.
