# Supplementary material for: Psychometric evaluation of the full and shortened versions of the WGCTA-II in Slovak university students
Source: Front Psychol. 2026 Feb 2;17:1764712. doi: 10.3389/fpsyg.2026.1764712 (PMC12910364; doi:10.3389/fpsyg.2026.1764712)
Supplement: Supplementary file 1 [file Table_1.docx]

Supplementary Material

**Supplementary Table 1.** Percentage of Correct Responses for Individual WGCTA-II Items

| **Item** | **% Correct** | **Item** | **% Correct** |
| --- | --- | --- | --- |
| WG_inference_1 | 31.06 | WG_inference_2 | 70.08 |
| WG_inference_3 | 33.71 | WG_inference_4 | 44.70 |
| WG_inference_5 | 38.64 | WG_inference_6 | 36.36 |
| WG_inference_7 | 46.97 | WG_inference_8 | 40.91 |
| WG_inference_9 | 56.06 | WG_inference_10 | 57.95 |
| WG_inference_11 | 29.17 | WG_inference_12 | 42.42 |
| WG_inference_13 | 15.91 | WG_inference_14 | 35.61 |
| WG_inference_15 | 50.76 | WG_inference_16 | 10.98 |
| WG_assumptions_17 | 66.67 | WG_assumptions_18 | 29.55 |
| WG_assumptions_19 | 74.62 | WG_assumptions_20 | 74.62 |
| WG_assumptions_21 | 89.39 | WG_assumptions_22 | 48.48 |
| WG_assumptions_23 | 81.06 | WG_assumptions_24 | 15.53 |
| WG_assumptions_25 | 66.67 | WG_assumptions_26 | 75.38 |
| WG_assumptions_27 | 71.59 | WG_assumptions_28 | 87.88 |
| WG_assumptions_29 | 60.23 | WG_assumptions_30 | 69.32 |
| WG_assumptions_32 | 56.82 | WG_deduction_33 | 63.64 |
| WG_deduction_34 | 66.29 | WG_deduction_35 | 23.48 |
| WG_deduction_36 | 67.05 | WG_deduction_37 | 49.62 |
| WG_deduction_38 | 38.64 | WG_deduction_39 | 65.91 |
| WG_deduction_40 | 74.62 | WG_deduction_41 | 54.55 |
| WG_deduction_42 | 41.67 | WG_deduction_43 | 45.45 |
| WG_deduction_44 | 34.85 | WG_deduction_45 | 48.86 |
| WG_deduction_46 | 62.88 | WG_deduction_47 | 55.68 |
| WG_deduction_48 | 53.03 | WG_interpretation_49 | 53.03 |
| WG_interpretation_50 | 64.77 | WG_interpretation_51 | 71.21 |
| WG_interpretation_52 | 41.29 | WG_interpretation_53 | 84.85 |
| WG_interpretation_54 | 87.88 | WG_interpretation_55 | 73.86 |
| WG_interpretation_56 | 70.08 | WG_interpretation_57 | 65.91 |
| WG_interpretation_58 | 33.33 | WG_interpretation_59 | 29.55 |
| WG_interpretation_60 | 81.44 | WG_interpretation_61 | 70.08 |
| WG_interpretation_62 | 42.05 | WG_interpretation_63 | 53.79 |
| WG_interpretation_64 | 17.42 | WG_arguments_65 | 65.15 |
| WG_arguments_66 | 79.17 | WG_arguments_67 | 76.89 |
| WG_arguments_68 | 74.24 | WG_arguments_69 | 77.27 |
| WG_arguments_70 | 67.80 | WG_arguments_71 | 77.65 |
| WG_arguments_72 | 37.88 | WG_arguments_73 | 53.41 |
| WG_arguments_74 | 70.83 | WG_arguments_75 | 49.24 |
| WG_arguments_76 | 53.41 | WG_arguments_77 | 57.58 |
| WG_arguments_78 | 64.77 | WG_arguments_79 | 62.50 |
| WG_arguments_80 | 62.88 | — | — |

**Supplementary Table 2.** Standardized factor loadings for WGCTA dimensions

| **Dimension** | **Item** | **Factor loading (λ)** |
| --- | --- | --- |
| **Inference** | WG_inference_1 | 0.282 |
|  | WG_inference_2 | 0.616 |
|  | WG_inference_3 | 0.001 |
|  | WG_inference_4 | 0.245 |
|  | WG_inference_5 | 0.484 |
|  | WG_inference_6 | 0.260 |
|  | WG_inference_7 | 0.236 |
|  | WG_inference_8 | 0.317 |
|  | WG_inference_9 | 0.507 |
|  | WG_inference_10 | 0.543 |
|  | WG_inference_11 | 0.115 |
|  | WG_inference_12 | 0.318 |
|  | WG_inference_13 | -0.003 |
|  | WG_inference_14 | 0.144 |
|  | WG_inference_15 | 0.499 |
|  | WG_inference_16 | 0.100 |
| **Assumptions** | WG_assumptions_17 | 0.099 |
|  | WG_assumptions_18 | 0.509 |
|  | WG_assumptions_19 | 0.196 |
|  | WG_assumptions_20 | -0.088 |
|  | WG_assumptions_21 | 0.269 |
|  | WG_assumptions_22 | -0.063 |
|  | WG_assumptions_23 | 0.159 |
|  | WG_assumptions_24 | -0.112 |
|  | WG_assumptions_25 | 0.398 |
|  | WG_assumptions_26 | -0.161 |
|  | WG_assumptions_27 | 0.259 |
|  | WG_assumptions_28 | 0.257 |
|  | WG_assumptions_29 | 0.350 |
|  | WG_assumptions_30 | 0.956 |
|  | WG_assumptions_32 | -0.057 |
| **Deduction** | WG_deduction_33 | 0.448 |
|  | WG_deduction_34 | -0.121 |
|  | WG_deduction_35 | -0.006 |
|  | WG_deduction_36 | -0.020 |
|  | WG_deduction_37 | -0.718 |
|  | WG_deduction_38 | -0.851 |
|  | WG_deduction_39 | 0.118 |
|  | WG_deduction_40 | -0.073 |
|  | WG_deduction_41 | -0.460 |
|  | WG_deduction_42 | 0.396 |
|  | WG_deduction_43 | -0.133 |
|  | WG_deduction_44 | 0.106 |
|  | WG_deduction_45 | 0.404 |
|  | WG_deduction_46 | -0.351 |
|  | WG_deduction_47 | 0.492 |
|  | WG_deduction_48 | 0.539 |
| **Interpretation** | WG_interpretation_49 | 0.390 |
|  | WG_interpretation_50 | 0.074 |
|  | WG_interpretation_51 | 0.691 |
|  | WG_interpretation_52 | -0.203 |
|  | WG_interpretation_53 | 0.601 |
|  | WG_interpretation_54 | 0.622 |
|  | WG_interpretation_55 | 0.327 |
|  | WG_interpretation_56 | 0.634 |
|  | WG_interpretation_57 | -0.080 |
|  | WG_interpretation_58 | 0.055 |
|  | WG_interpretation_59 | 0.337 |
|  | WG_interpretation_60 | 0.589 |
|  | WG_interpretation_61 | 0.538 |
|  | WG_interpretation_62 | 0.399 |
|  | WG_interpretation_63 | 0.310 |
|  | WG_interpretation_64 | 0.036 |
| **Arguments** | WG_arguments_65 | 0.299 |
|  | WG_arguments_66 | 0.409 |
|  | WG_arguments_67 | 0.381 |
|  | WG_arguments_68 | 0.414 |
|  | WG_arguments_69 | 0.590 |
|  | WG_arguments_70 | 0.311 |
|  | WG_arguments_71 | 0.587 |
|  | WG_arguments_72 | 0.133 |
|  | WG_arguments_73 | 0.210 |
|  | WG_arguments_74 | 0.133 |
|  | WG_arguments_75 | 0.258 |
|  | WG_arguments_76 | 0.410 |
|  | WG_arguments_77 | 0.010 |
|  | WG_arguments_78 | 0.495 |
|  | WG_arguments_79 | 0.387 |
|  | WG_arguments_80 | 0.385 |
